# Characterization of an ester-based core-multishell (CMS) nanocarrier for the topical application at the oral mucosa

**DOI:** 10.1007/s00784-021-03884-x

**Published:** 2021-04-05

**Authors:** H. Dommisch, KN. Stolte, J. Jager, K. Vogel, R. Müller, S. Hedtrich, M. Unbehauen, R. Haag, K. Danker

**Affiliations:** 1 Department of Periodontology, Oral Medicine and Oral Surgery, Charité - Universitätsmedizin Berlin, corporate member of Freie Universität Berlin, Humboldt-Universität zu Berlin, and Berlin Institute of Health, 14197 Berlin, Germany; 2 Department of Periodontology, University of Washington, Seattle, WA USA; 3 Institute for Biochemistry, Charité - Universitätsmedizin Berlin, corporate member of Freie Universität Berlin, Humboldt-Universität zu Berlin, and Berlin Institute of Health, 10117 Berlin, Germany; 4 Pharmacology and Toxicology, Institute of Pharmacy, Freie Universität Berlin, 14195 Berlin, Germany; 5 Faculty of Pharmaceutical Sciences, University of British Columbia, 2405 Wesbrook Mall, Vancouver, BC V6T1Z3 Canada; 6 Institute for Chemistry and Biochemistry, Freie Universität Berlin, 14195 Berlin, Germany

**Keywords:** Core-multishell nanocarrier, Transepithelial resistance, Penetration, Oral mucosal equivalents

## Abstract

**Objectives:**

Topical drug administration is commonly applied to control oral inflammation. However, it requires sufficient drug adherence and a high degree of bioavailability. Here, we tested the hypothesis whether an ester-based core-multishell (CMS) nanocarrier is a suitable nontoxic drug-delivery system that penetrates efficiently to oral mucosal tissues, and thereby, increase the bioavailability of topically applied drugs.

**Material and methods:**

To evaluate adhesion and penetration, the fluorescence-labeled CMS 10-E-15-350 nanocarrier was applied to ex vivo porcine masticatory and lining mucosa in a Franz cell diffusion assay and to an in vitro 3D model. In gingival epithelial cells, potential cytotoxicity and proliferative effects of the nanocarrier were determined by MTT and sulphorhodamine B assays, respectively. Transepithelial electrical resistance (TEER) was measured in presence and absence of CMS 10-E-15-350 using an Endohm-12 chamber and a volt-ohm-meter. Cellular nanocarrier uptake was analyzed by laser scanning microscopy. Inflammatory responses were determined by monitoring pro-inflammatory cytokines using real-time PCR and ELISA.

**Results:**

CMS nanocarrier adhered to mucosal tissues within 5 min in an in vitro model and in ex vivo porcine tissues. The CMS nanocarrier exhibited no cytotoxic effects and induced no inflammatory responses. Furthermore, the physical barrier expressed by the TEER remained unaffected by the nanocarrier.

**Conclusions:**

CMS 10-E-15-350 adhered to the oral mucosa and adhesion increased over time which is a prerequisite for an efficient drug release. Since TEER is unaffected, CMS nanocarrier may enter the oral mucosa transcellularly.

**Clinical relevance:**

Nanocarrier technology is a novel and innovative approach for efficient topical drug delivery at the oral mucosa.

**Supplementary Information:**

The online version contains supplementary material available at 10.1007/s00784-021-03884-x.

## Introduction

In the oral cavity, the epithelial surface is constantly exposed to high numbers of not only highly variable microorganisms, but also chemical, thermal, and mechanical environmental factors. The epithelial tissues exhibit effective physical as well as chemical defense properties that prevent infection and physical tissue damage. In some cases, however, this robust epithelial barrier may be more vulnerable and develop various inflammatory disease characteristics.

Periodontitis is a highly prevalent bacterially induced inflammatory disease along with epithelial barrier and bone tissue breakdown [1–3]. Besides mechanical treatment strategies, systemic antibiotics have been implemented to control the subgingival microbial flora. The adjunctive application of systemic antibiotics led to the reduction of periodontal pockets and inflammation [4, 5]. However, only a few patients with severe periodontitis benefit from intake of adjunctive systemic antibiotics when disease progression was analyzed, and the overall clinical effect was rather marginal [6]. Furthermore, the resistance of subgingival microflora against antibiotics is increased, if systemic antibiotics are frequently administered in a population [7, 8]. In addition, drug-mediated anti-inflammatory periodontal therapy is under current scientific discussion, and on the experimental level, promising approaches have been described [9, 10].

In addition to periodontitis, numerous other inflammatory conditions may, even simultaneously, be present at mucosal sites in the oral cavity. Lichen planus, Pemphigus vulgaris, and bullous mucosal pemphigoid not only are auto-immune diseases that are not restricted to the skin, but also show their characteristics on the oral mucosa [11]. Here, administration of topical drug formulations (e.g., cortisone) in form of cremes and mouth rinses is considered the standard therapeutical approach [12].

Topical application of drugs offers non-invasive feasibility, less side effects compared to oral or intravenous application, improved patient compliance, and increased bioavailability by avoiding the hepatic first-pass effect. However, topical application of any antiseptic and/or anti-inflammatory formulation is of reduced effectiveness for the patient due to limited bioavailability of current topical treatment approaches, time of application, and modes of application [13]. In general, many drugs show weak adherence to oral mucosal surfaces as well as in periodontal mucosal pockets due the constant flow of saliva (on average, 500-600 ml/day), mechanical drug displacement during chewing, and constant exudation of gingival crevicular fluid, a serum-like exudate secreted into the periodontal space [14–16].

Therefore, there is a strong medical need for novel therapeutic strategies for the treatment of periodontitis and other inflammatory diseases of the oral mucosa that combine a high grade of drug bioavailability and a safe regimen. Nanocarrier technology has the potential to overcome issues regarding bioavailability of any drug applied at mucosal sites. Even the special inflammatory environment may be utilized to increase the efficiency of application using photo-triggered, pH-triggered, protease-triggered, or temperature-triggered release mechanisms [17–22].

**Fig. 1:**
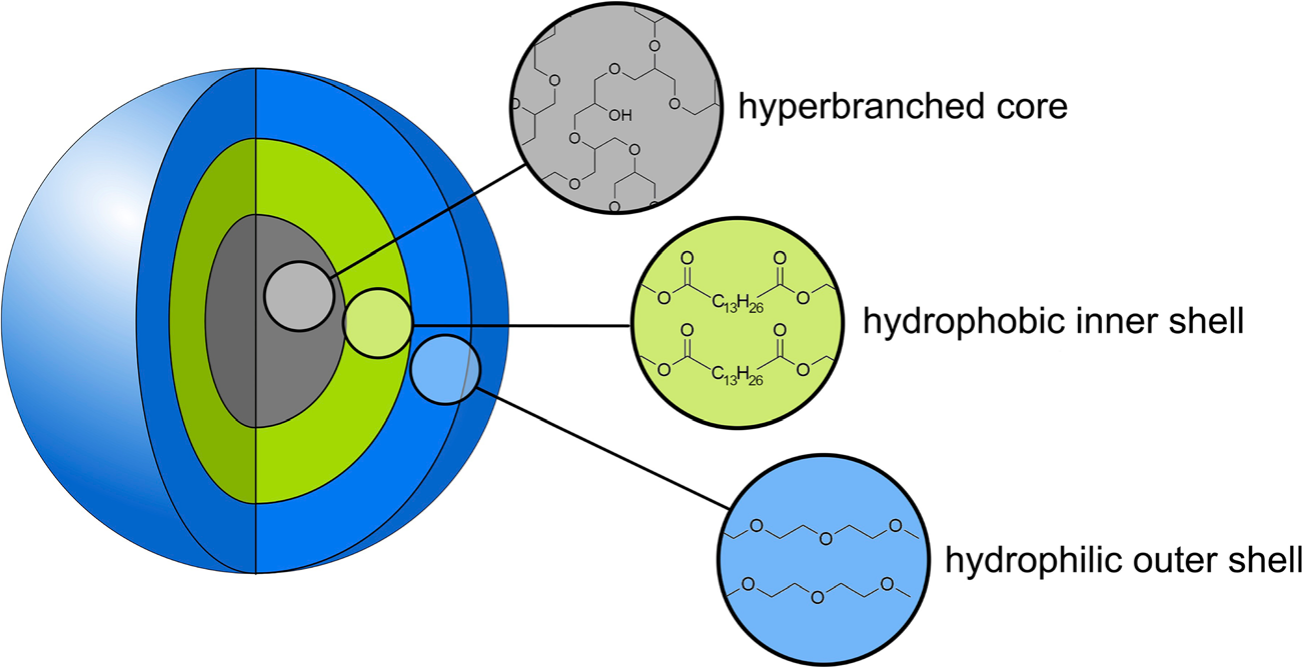
Schematic representation of the dendritic core-multishell nanocarrier [24]. This type of nanocarrier allows loading of hydrophilic as well as of hydrophobic drugs and topical delivery at sites of inflammation

Recently, we introduced the concept of core-multishell (CMS) nanocarrier as a novel carrier system to increase bioavailability of topically applied dexamethasone at the oral mucosa [23] (Fig. 1). The hPG-amide-C18-PEG-core-multishell nanocarrier (CMS 10-A-18-350) was applied to different porcine mucosal surfaces and showed excellent penetration properties [23]. In an ex vivo approach, we were able to show that dexamethasone loaded to a CMS nanocarrier was more efficiently released and taken up by oral mucosal tissues compared to dexamethasone from a conventional cream formulation [23]. However, it has been shown that application of the CMS 10-A-18-350 nanocarrier exhibited cytotoxic effects at high concentrations and after longer exposure times [23].

In the present study we, therefore, analyzed a novel generation of biodegradable, ester-based CMS nanocarriers. One of these newly developed nanocarriers, the CMS 10-E-15-350, was identified as the most biocompatible carrier in different models [25]. Inskin, the CMS 10-E-15-350 was identified as the most promising carrier [25]. Therefore, we chose and evaluated this specific nanocarrier for its ability to adhere and penetrate into oral mucosal tissues.

We hypothesized that the ester-based CMS 10-E-15-350 shows efficient mucosal penetration properties without inflammatory, cytotoxic or proliferative side effects. We found that penetration of CMS 10-E-15-350 can be observed within minutes after mucosal application. We did not detect any influence on proliferation and the metabolic activity. Application of the nanocarrier did not provoke an inflammatory response of gingival epithelial cells and the physical barrier was not altered since the transepithelial electrical resistance remained unaffected. Therefore, we expect excellent biocompatibility of this nanocarrier generation in future studies.

## Material and methods

### Core-multishell nanocarrier 10-E-15-350

The synthesis of the CMS 10-E-15-350 nanocarrier was recently described in [25]. The nanocarrier was soluble in culture medium. Dynamic light scatting measurements revealed that 71% of the nanocarrier particles solution had a size of 22.5nm.

For penetration and uptake experiments, the respective fluorescence-labeled version of the CMS ester-based nanocarrier (CMS 10-cICC-E-15-350, DendroPharm, Berlin, Germany) was used at a concentration of 10mg/ml. As a fluorescence label, the dye indocarbocyanine (ICC) was utilized. For all other experiments, the non-labeled carrier was used (Fig. 1).

### Tissue and cell culture

For ex vivo experiments, the untreated native mucosa was dissected from areas of both masticatory and lining mucosa obtained from pigs slaughtered for food industry reasons. Tissues were then immediately stored in PBS for transportation and subjected to Franz cell experiments as recently described [23]. For experiments with cell monolayers, the immortalized human gingival keratinocytes OKG4/bmi1/TERT (OKG4; kindly provided by Susan Gibbs, Amsterdam) were used [26, 27]. Primary gingival epithelial cells (GECs) were dissected from gingival tissue samples obtained from tooth extractions, and tissues were and kindly provided by the Department of Oral and Maxillofacial Surgery at Campus Virchow, Charité.

Keratinocytes were cultured in DermaLife K medium with 1% penicillin/streptomycin in the presence of 60 μM or 1.4 mM Ca^2+^ as indicated (CellSystems, Troisdorf, Germany). Cells were cultivated in collagen-coated plates and flasks at 37°C and 5% CO_2_.

### Franz cell experiments

Penetration studies were performed as recently described [23]. Porcine oral mucosal tissues (2 cm diameter) were mounted onto static-type Franz cells (diameter 7 mm, volume 5 ml, PermeGear, Bethlehem, PA, USA), and the nanocarrier was applied onto the mucosal surface for the time points indicated. Microsections of PFA-embedded tissues were analyzed for nanocarrier adhesion and penetration using the confocal laser scanning microscope LSM700MAT (CLSM, Zeiss). Three independent experiments were performed using three biological replicates for both masticatory and lining mucosa at each time point. More detailed information is given in the Supplementary information.

### 3D culture of in vitro organotypic mucosal equivalents

To establish a model for tracking nanocarrier penetration within the oral mucosal tissue, a 3D organotypic cell culture was established [28]. Here, OKG4 cells and the fibroblast cell line (T0026) were co-cultured.

Briefly, 1 ml of a collagen I solution mixed with 4 × 10^5^ cells/ml gingival fibroblasts was transferred on an acellular collagen sheet from bovine type I collagen (0.77 mg/ml; Nutragen^®^, Advanced BioMatrix) in a Millicell^®^ culture plate insert (30 mm diameter, pore size 0.4 μm; Merck). Inserts were placed in 6-well plates and incubated at 37°C and 5% CO_2_ for 1 h. Prior to the addition of OKG4 cells (1 × 10^6^, DermaLife K medium, 60 μM Ca^2+^, Lifeline Cell Technology), fibroblast-populated collagen gels were cultured for 5 days in DMEM (Corning) containing 10% FCS (PAN-Biotech). For more detailed information, please see the Supplementary information.

### Cell viability assays

Assays for the determination of cell viability were recently described by our group [23]. Briefly, MTT and sulphorhodamine B (SRB) assays were performed upon nanocarrier exposure to OKG4 cells. Cell viability was monitored for 24, 48, and 72 h. A total of three experiments were performed with each consisted of six technical replications. For further analysis, mean values of untreated control cells were set at 100% in each of the three experiments. Test groups were normalized to untreated control cells. Graphs represent the three experimental sets, and error bars indicate standard deviations in the test groups when normalized to controls. Values > 80% predicted no cytotoxic effects. More detailed information is given in the supplementary electronic file (Supplementary information Figure 1 A,B).

### Measurement of the transepithelial electrical resistance

For the measurement of the transepithelial electrical resistance (TEER), 12 mm Transwell^®^ inserts with 0.4 μm pore polycarbonate membranes (Corning) coated with collagen IV (20 μg/ml, Sigma-Aldrich) were used. OKG4 cells (2.7 × 10^4^) in 500 μl in DermaLife K medium containing 60 μM Ca^2+^ were seeded to the filter, and the medium was changed every 1–3 days. When cells reached confluence (approx. after 7 days), they were cultured in a medium containing 1.4 mM Ca^2+^ to induce terminal cell differentiation [29] and the formation of tight junctions [30]. Subsequently, 50 μg/ml of the nanocarrier solution was applied to the cells. As a control, the TEER of cells that were kept in DermaLife K medium containing 60 μM Ca^2+^ was determined. The TEER values were measured using an Endohm-12 chamber (World Precision Instruments) and a volt-ohm-meter (Millipore). After subtracting the blank filter’s TEER, the value was multiplied by the filter area (1.12 cm^2^). Four independent experiments were performed in duplicate.

### Cellular uptake

The 5 × 10^4^ OKG4 cells/well in DermaLife medium containing 60 μM Ca^2+^ were seeded to 8-well Permanox slides (Nunc) and cultured for 24h. The cells were subsequently cultivated in 60 μM or 1.4 mM Ca^2+^ for further 24 h and treated with the respective cell culture medium containing ICC-coupled CMS 10-E-15-350 at a final ICC concentration of 2 μg/ml for further 24 h. The cell nuclei were stained with Hoechst 33342 dye (blue, Invitrogen) and cell membranes were stained with Wheat Germ Agglutinin (WGA) Alexa Fluor 488-conjugated (Invitrogen), which binds to N-acetylglucosamine and N-acetylneuraminic acid moieties on the cell surface, for 30 min. Subsequently, the cells were rinsed with PBS to remove the excess of dyes. The internalization was observed using the LSM700MAT (Zeiss) and analyzed by the ZEN software (Zeiss). Each analysis was performed in triplicate.

### qPCR and ELISA experiments

Detailed information on the methodology of qPCR and ELISA experiments is displayed in the Supplementary information.

### Statistical analysis

For statistical analyses (Graph Pad Prism; *P* < 0.05), absolute values were calculated using one-way ANOVA corrected by the Dunnett’s multiple comparison test and the Tukey’s multiple comparison test. For SRB and MTT assays, mean values determined from control cells were set at 100% as reference for test groups. The figures display relative values given in % (bars) with standard deviation (errors bars), and analysis was performed using the Holm-Sidak´s multiple comparison test. Real-time PCR experiments were analyzed using the one-way ANOVA with correction for multiple testing (Dunnett’s multiple comparison test). For mRNA expression analysis, only relative fold changes greater than 2-fold were considered biologically relevant (marked by a dashed line). A *P*-value of *P* < 0.05 was considered significant.

## Results

### Time-dependent penetration and adherence of CMS nanocarriers in porcine mucosal tissues

**Fig. 2:**
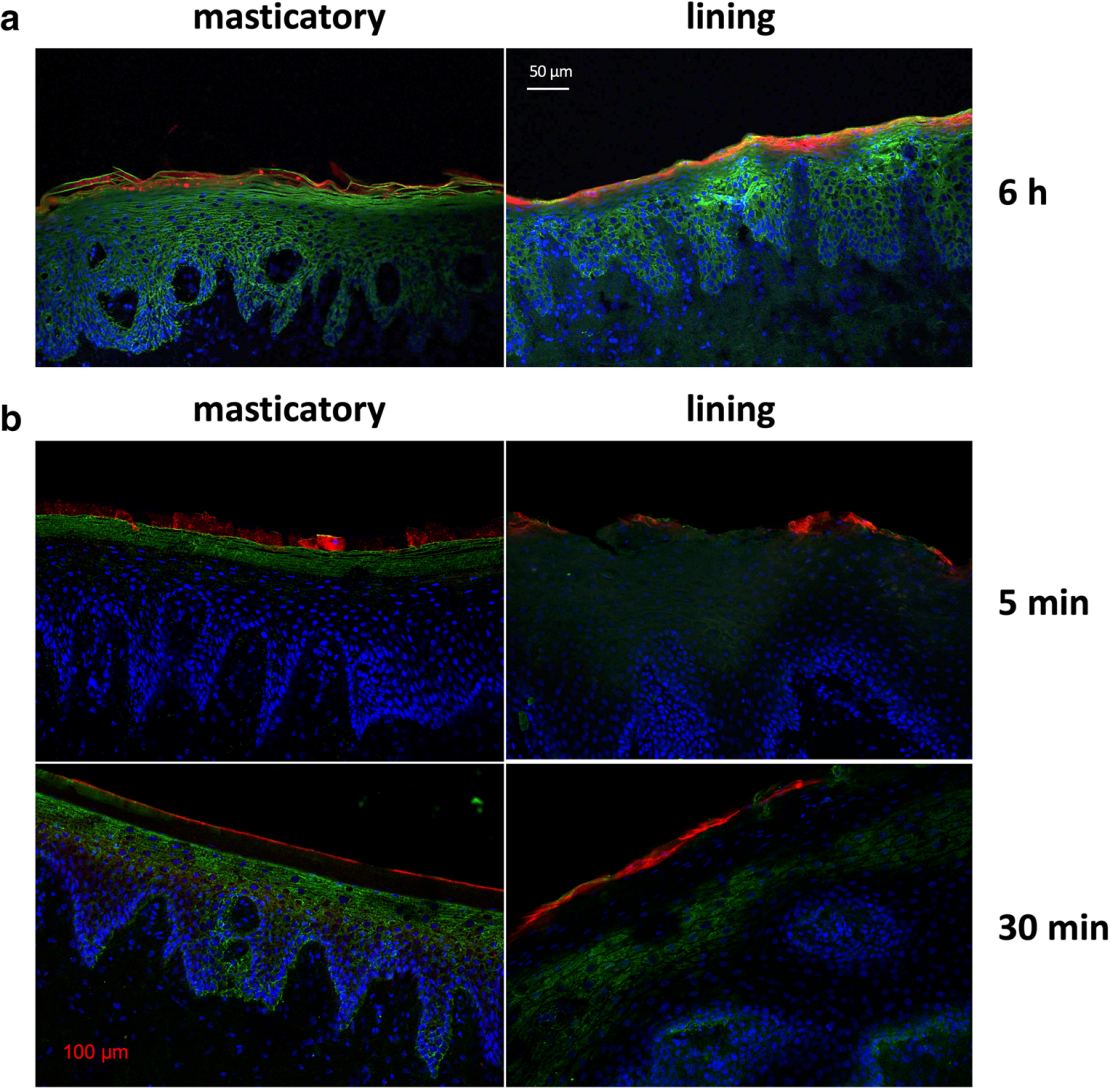
Representative images of nanocarrier penetration into ex vivo masticatory and lining porcine mucosa at various time points (**a**, **b**). Microsections were stained for pan-cytokeratin shown in green and cell nuclei visualized with the Hoechst 33342 dye (blue). The CMS 10-E-15-350 nanocarrier was labeled with the fluorescent dye indocarbocyanine (red). Magnification ×20

Penetration of the CMS 10-E-15-350 nanocarrier coupled to a fluorescent dye was analyzed using porcine masticatory and lining mucosa and confocal microscopy (Fig. 2a). After 6 h, the nanocarrier penetrated into both mucosal tissue types (Fig. 2a). While the CMS nanocarrier penetrated evenly into the para-keratinized lining mucosa, it was prone to aggregation in the *stratum corneum* of the masticatory mucosa.

For clinical translation, shorter application times were tested. After 5 min, the nanocarrier showed a sparse penetration pattern in the superior cell layer of the lining mucosa, whereas at the masticatory mucosa, the nanocarrier exhibited firm adhesion with no evidence for penetration. After 30 min, the CMS nanocarrier penetrated into the lining mucosa with a comparable depth observed for the 6-h time point, while the majority of the nanocarrier only adhered to the *stratum corneum* of the masticatory mucosa (Fig. 2b).

### 3D culture technique as a novel experimental design to track nanocarrier penetration

**Fig. 3:**
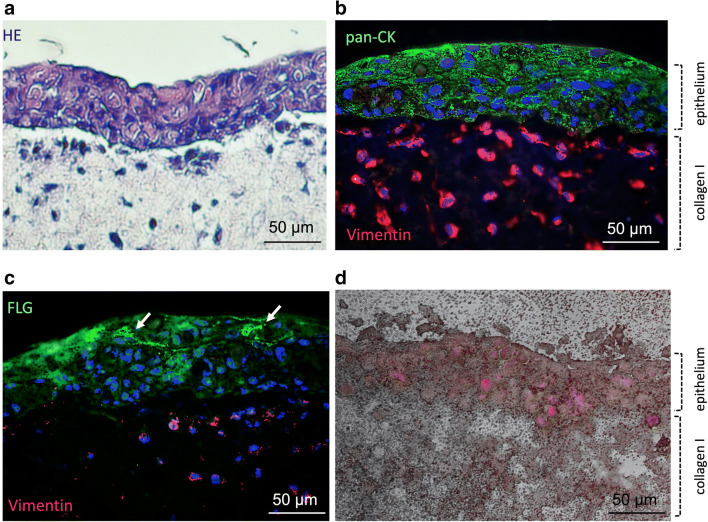
Representative images of nanocarrier penetration into in vitro 3D mucosal equivalents of co-cultured gingival keratinocytes and collagen/fibroblast gel. **a** Microsections were stained for hematoxylin-eosin. **b** Pan-cytokeratin (green) and vimentin (red). **c** Filaggrin and vimentin. Cell nuclei were visualized with the Hoechst 33342 dye (blue). **d** Microscopic image of the penetrated nanocarrier coupled to the fluorescent dye indocarbocyanine (red). Magnification ×40

Additionally, we tested the penetration of the CMS 10-E-15-350-ICC nanocarrier in an organotypic 3D model (Fig. 3). The multi-layered epidermal component was anchored to the underlying collagen/fibroblast gel (Fig. 3a). The epidermal cells expressed keratins (green) and the fibroblasts were positive for vimentin (red; Fig. 3b). The epidermal component revealed clear signs of terminal differentiation since cells of the upper part expressed the differentiation marker filaggrin (Fig. 3c). Cells of the basal layer showed round cell bodies and nuclei, while epithelial cells from the upper layer changed their morphology to a plainer shape of cells and nuclei (Fig. 3 a and b).

Nanocarrier application to the surface of the 3D model exhibited penetration into the epidermal compartment after 5 min. The collagen matrix underneath the epithelial cell layer was not penetrated by the nanocarrier (red, Fig. 3d).

### CMS 10-E-15-350 nanocarrier did not cause cytotoxic effects in OKG4 cells

**Fig. 4:**
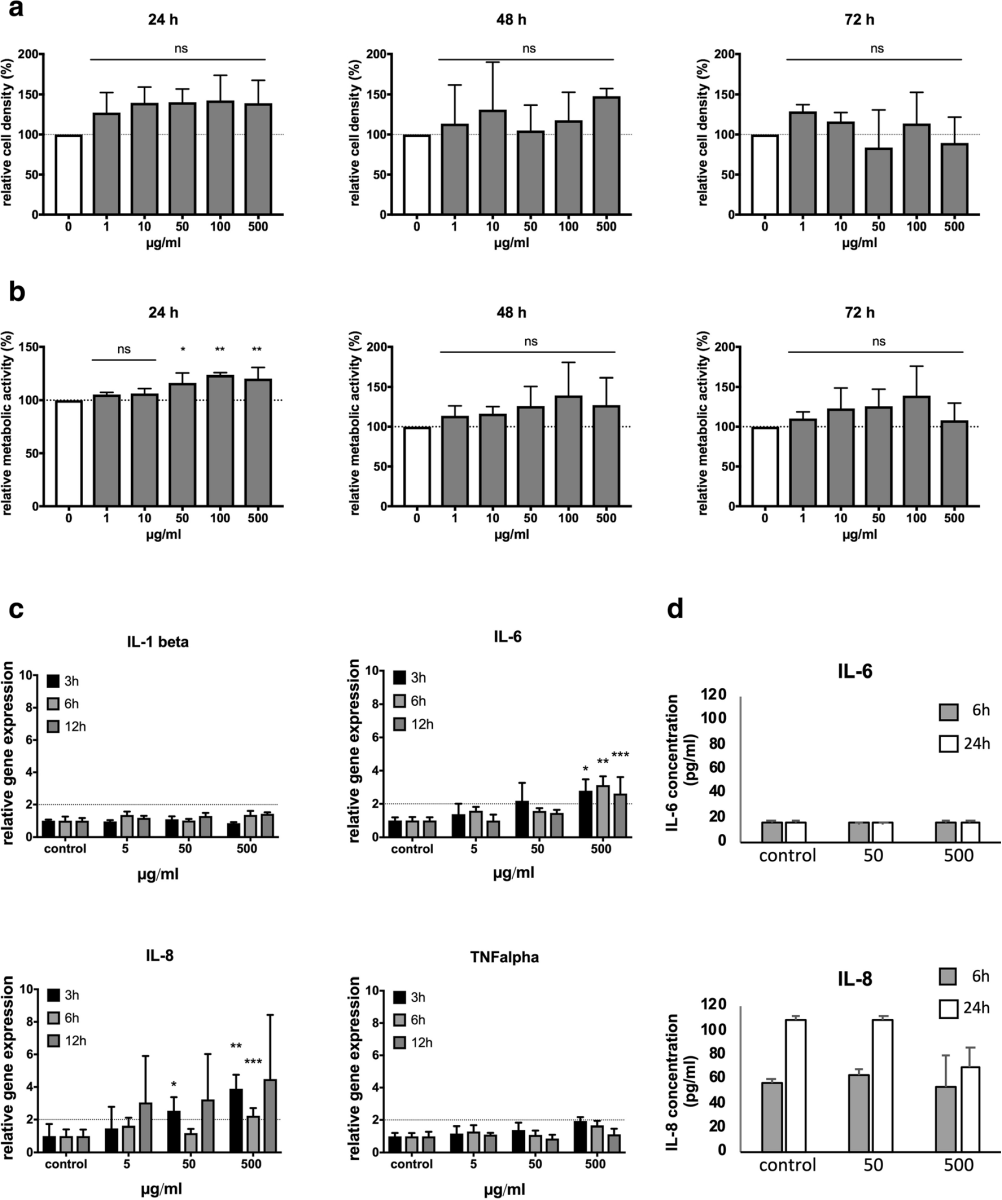
**a** Cell proliferation assay (sulphorhodamine B). OKG4 cells were exposed to nanocarrier concentrations of 1, 10, 50, 100, and 500 μg/ml for 24, 48, and 72 h. **b** Measurement of the metabolic activity (MTT) of OKG4 cell upon application of the CMS 10-E-15-350 nanocarrier under the afore mentioned conditions. * *P* = 0.0182; ** *P* = 0.0021. **c** Analysis of the mRNA expression of IL-1 beta, IL-6 (* *P* = 0.0103; ** *P* = 0.0001; *** *P* = 0.0001), IL-8 (* *P* = 0.0416; ** *P* = 0.0002; *** *P* = 0.0002), and TNF alpha upon nanocarrier application on gingival keratinocytes (OKG4). Gene expression analysis was performed by real-time PCR experiments. **d** Cytokine secretion analyses of IL-6 and IL-8 using ELISA technology

For each time point tested, cell proliferation of immortalized gingival keratinocytes was not affected by the nanocarrier compared to untreated control cells (Fig. 4a).

After 24 h, the metabolic activity was increased by a nanocarrier concentrations of 50 μg/ml, 100 μg/ml, and 500 μg/m. After 48 and 72 h, an influence on the metabolic activity of cells could not be observed at any concentration tested (Fig. 4b). In primary gingival keratinocytes, an influence on the metabolic activity in the presence of 50 and 500 μg/ml CMS 10-E-15-350 could not be observed (Supplementary information Figure 2 A, B).

### CMS 10-E-15-350 did not affect the secretion of pro-inflammatory cytokines

Gene expression analyses revealed that the mRNAs of the pro-inflammatory cytokines IL-1 beta and TNF alpha were not altered upon nanocarrier exposure regardless of the concentration and the application time (Fig. 4c). The mRNA of IL-6 was upregulated by the nanocarrier at the highest concentration (500 μg/ml) applied after 3, 6, and 12 h compared to corresponding control cells (Fig. 4d). For IL-8, the nanocarrier caused an upregulation of mRNA expression at concentrations of 50 μg/ml after 3 h and of 500 μg/ml after 3 h and 6 h (Fig. 4c). However, analysis of the medium of cells treated with 50 and 500 μg/ml CMS 10-E-15-350 revealed that the concentration of IL-6 and IL-8 was not altered compared control cells (Fig. 4d).

### Transepithelial electrical resistance of OKG4 cells remained unchanged in the presence of CMS nanocarriers

**Fig. 5:**
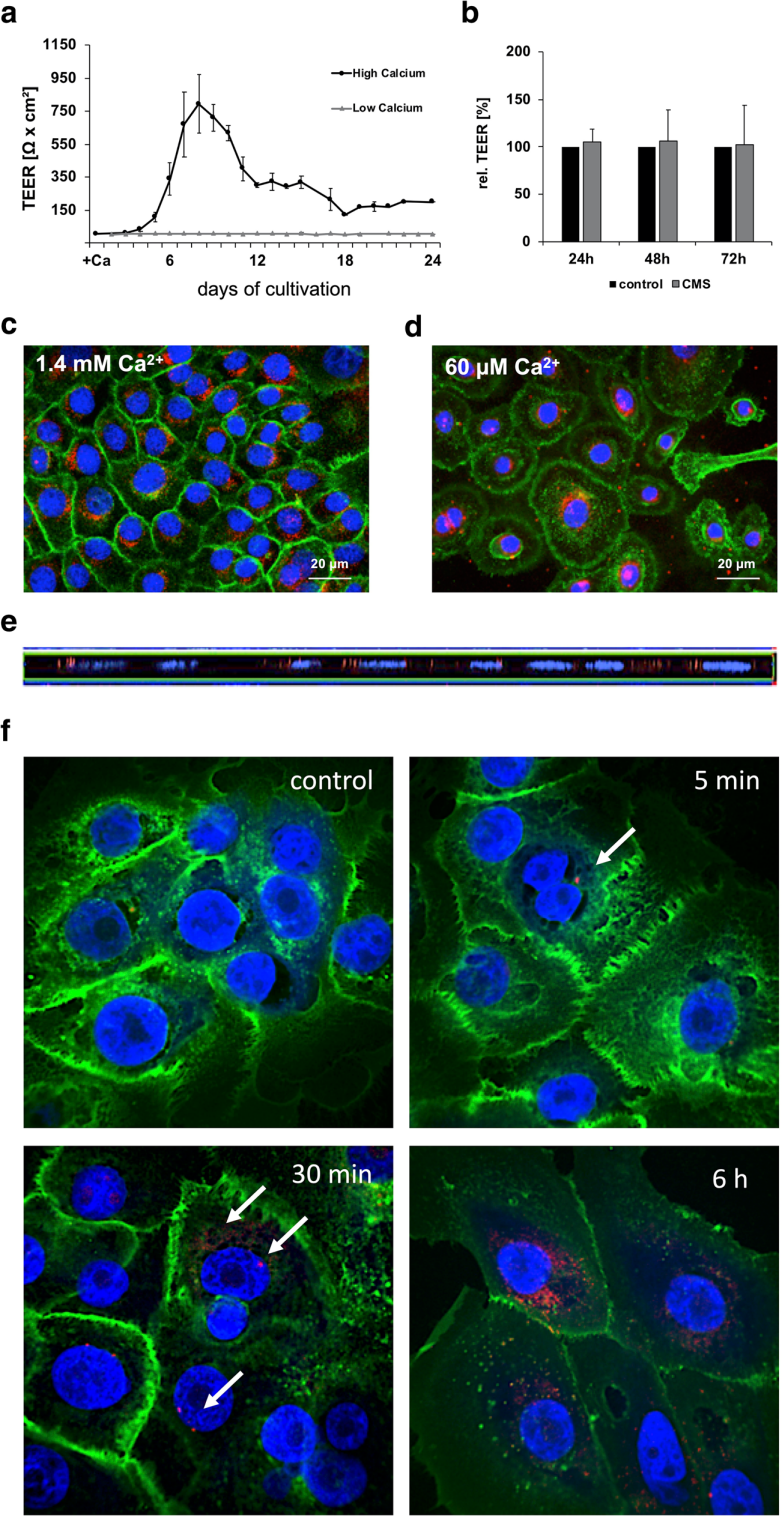
**a** Measurement of transepithelial electrical resistance (TEER) using OKG4 cells cultured in the presence of Ca^2+^ concentrations of 60 μM (gray line) and 1.4 mM (black line), respectively. **b** Relative TEER-measurements in OKG4 cell cultures when exposed to the CMS 10-E-15-350 nanocarrier (gray bars) compared to untreated control cells (black bars) for 24, 48, and 72 h. Visualization of nanocarrier uptake into epithelial cells (OKG4) stained with Alexa Fluor 488-coupled wheat germ agglutinin (WGA; green) and Hoechst 33342 dye (blue) in the presence of **c** 1.4 mM Ca^2+^ and **d** 60 μM. Intracellular perinuclear localization of the CMS 10-E-15-350 nanocarrier coupled to the fluorescent dye indocarbocyanine (red) was monitored by CLSM (magnification ×63), and **e** confirmed by an orthogonal projection. **f** Visualization of nanocarrier uptake into primary epithelial cells over time. Cells were incubated with indocarbocyanine-coupled CMS 10-E-15-350 nanocarrier (red) for 5 min, 30 min, and 6 h or left untreated. Subsequently, cells were stained with Alexa Fluor 488-coupled wheat germ agglutinin (WGA; green) and Hoechst 33342 dye (blue, magnification ×60)

To study the penetration route of the nanocarrier, we characterized the physical barrier formed by differentiating OKG4 cells (Fig. 5a-e). For this purpose, cells were grown to confluence in the presence of 60 μM Ca^2+^. Then, cell differentiation was induced by increasing the Ca^2+^ concentration in the medium to 1.4 mM (high Ca^2+^). Under these conditions, a constantly increasing TEER was monitored with a peak at day 8. Then, the resistance decreased but remained stable between day 12 and 24 (Fig. 5a). Cells cultivated in the presence of 60 μM did not exhibit a measurable resistance (Fig. 5a).

The increase in TEER was accompanied by formation of tight cell-cell contacts (Supplementary information Figure 3 A). Cells cultured in the presence of 60 μM Ca^2+^ (low Ca^2+^) showed no cell-to-cell contacts (Supplementary information Figure 3 B).

Administration of CMS 10-E-15-350 affected neither the TEER of the cell monolayer compared to control cells (Fig. 5b) nor the integrity of cell-cell contacts (Fig. 5 c, d, Supplementary information Figure 4). Using fluorescence analysis, we showed that the fluorescence-coupled nanocarrier (red) was taken up by differentiated (Fig. 5c) and undifferentiated cells (Fig. 5d). The counterstaining of cell nuclei with Hoechst 33342 (blue) revealed that the nanocarrier is localized perinuclearily (Fig. 5 c, d) as confirmed by orthogonal (xz) projections (Fig. 5e). In primary epithelial cells, low amounts of the CMS 10-E-15-350 were found even after 5 min in the cytosol. The amount of nanocarrier within the cells increased over time (Fig. 5f).

## Discussion

Limited bioavailability upon topical drug application still represents the major challenge for the treatment of oral inflammatory diseases. Here, we tested the hypothesis whether a new generation of an ester-based CMS nanocarrier, CMS 10-E-15-350, is a suitable nontoxic drug-delivery system that penetrates efficiently to oral mucosal tissues without influencing the inflammatory response, the metabolic activity, and the proliferation rate of gingival epithelial cells.

While the CMS nanocarrier penetrated relatively evenly into the para-keratinized lining mucosa, it was prone to aggregation in the *stratum corneum* of the masticatory mucosa. The penetration behavior of the CMS 10-E-15-350 nanocarrier was in accordance with the penetration observed for the CMS 10-A-18-350 nanocarrier into the oral mucosa [23]. However, this was contrary to skin experiments which revealed that CMS nanocarriers failed to penetrate into intact skin. Here, penetration was only observed into compromised skin [31].

In contrast to our previous study, shorter nanocarrier exposure times were chosen. After 30 min, the CMS nanocarrier penetrated into the lining mucosa to a comparable extent as observed after 6 h [23]. Even after 5 min, nanocarrier penetration was observed but to a lesser extent compared to the longer exposure times. These findings suggest that especially in mucosal types that exhibit lower degrees of keratinization, CMS nanocarriers may reach a sufficient penetration depth even after a short application time. A penetration time within minutes may allow an appropriate clinical handling with a higher degree of patient´s acceptance.

In general, ex vivo and/or in vivo animal experiments should be reduced or avoided whenever possible [32–34]. We have, therefore, established a novel experimental approach to monitor penetration dynamics of nanocarrier in a 3D full-thickness gingiva equivalent that is structurally comparable to human oral mucosal tissues. Similar to the ex vivo experiments, the nanocarrier penetrated through the epithelial cell layers, but not into the underlying connective tissue.

This approach will open new avenues for novel experimental designs that will facilitate research into drug delivery, release, and efficacy of anti-inflammatory compounds at the cellular and subcellular levels compared to in vivo and ex vivo experiments.

Our results showed that the nanocarrier CMS 10-E-15-350 did not interfere with the physical integrity of the mucosal tissue model. Penetration may occur by paracellular or transcellular routes. As determined by transepithelial electrical resistance (TEER) measurements, OKG4 cells developed a tight physical barrier that is comparable or superior to other similar gingiva culture models published so far [30, 35]. Nanocarrier treatment of cells had no impact on the cell monolayer’s resistance. Furthermore, the ICC-coupled nanocarrier was found intracellularly after administration using confocal microscopy. These results favor a transcellular route into the oral mucosa. Similar intracellular pathways have been described reflecting processes also involved in autophagy (endosome/exosome secretory pathway) [36]. For the Langerhans cell line XS52, it has been shown that CMS nanocarrier is not taken up by one exclusive pathway, but by micropinocytosis, caveolae-mediated endocytosis, and clathrin-mediated endocytosis [37]. In this study, a perinuclear localization of CMS 10-E-15-350 was determined by CLSM analyses. Whether the CMS nanocarrier utilizes similar or specific cellular uptake mechanisms needs to be determined in future investigations.

Biosafety is an important issue in biomedical nanotechnology. Thus, nanoparticles should be bioinert or biodegradable and their use should not cause toxic side effects [38, 39].

The ester-based CMS 10-E-15-350 nanocarriers have been designed to produce hyperbranched polyglycerol (hPG), alkyl diacids, and mPEG350 on degradation to improve biocompatibility over amide-based CMS nanocarriers [40]. We were able to show that the CMS 10-E-15-350 nanocarrier caused no significant changes in cell proliferation or metabolic activity in gingival epithelial cells and may therefore be more suitable for the application at the oral mucosa compared to the amide-based CMS 10-A-18-350 nanocarrier which exhibited cytotoxic effects at high concentrations and after long exposure times in gingival epithelial cells [23]. The CMS 10-E-15-350 nanocarrier increased the metabolic activity merely at high concentrations within the first 24 h. This early activity may occur in conjunction with the intracellular uptake of the nanocarrier which was observed after 24 h. These data are in line with studies performed in skin models which showed that the CMS 10-E-15-350 nanocarrier demonstrated excellent biocompatibility as assessed in comprehensive toxicological assays [25].

Furthermore, it was demonstrated that the mRNA expression of IL-6 and IL-8 was upregulated only at the highest nanocarrier concentration (500 μg/ml) applied. The mRNA expression of the other pro-inflammatory mediators IL-1 beta, CCL-20, and TNF alpha remained unaffected. This suggests that CMS 10-E-15-350 nanocarrier should be applied at lower concentrations.

In conclusion, the present study introduced the newly developed core-multi shell nanocarrier CMS 10-E-15-350 to oral mucosal models and cells for the first time. This nanocarrier revealed fast adherence and penetration properties into the epithelial cell layer of oral mucosal tissues. Within the limitations of this study, the results of in vitro experiments indicated a transcellular penetration pathway into the epithelial tissue, and the transepithelial resistance, which is an indicator of the integrity of the physical barrier, remained unchanged. Physiological CMS concentrations did not provoke alterations in the cellular metabolic activity, the cellular proliferative status, or the immune response. Thus, this biodegradable nanocarrier may be considered biologically safe regarding its application onto oral mucosal tissues. Since this nanocarrier possesses the capability to encapsulate immunologically relevant drugs, such as dexamethasone and eternacept [17, 25], this technology opens new avenues for promising therapeutic approaches in the context of translational science for the treatment of oral diseases.

## Supplementary information

ESM 1 (DOCX 37 kb)

ESM 2 (JPG 149 kb)

ESM 3 (JPG 294 kb)

ESM 4 (JPG 1317 kb)

ESM 5 (JPG 171 kb)

## Declarations

All experiments followed the guidelines of good clinical/laboratory practice (GCP/GLP) and the WHO Declaration of Helsinki 1964, latest update Seoul 2008 (59th WMA General Assembly, Seoul, October 2008). The study was approved by the ethical committee of the Charité – Universitätsmedizin Berlin (IRB number: EA2/185/16). All volunteers gave their informed consent.
