# Supplementary material for: Characterization of an ester-based core-multishell (CMS) nanocarrier for the topical application at the oral mucosa
Source: Clin Oral Investig. 2021 Apr 5;25(10):5795–805. doi: 10.1007/s00784-021-03884-x (PMC8443517; doi:10.1007/s00784-021-03884-x)
Supplement: Supplementary file 1 — (DOCX 37 kb) [file 784_2021_3884_MOESM1_ESM.docx]

**Electronic supplementary file**

**Characterization of an ester-based core-multishell (CMS) nanocarriers for the topical application at the oral mucosa.**

Dommisch H^1,2*#^, Stolte KN^3#^, Jager J^3^, Vogel K^1^, Müller R^1^, Hedtrich S^4,5^, Unbehauen M^6^, Haag R^6^, Danker K^3^.

^1^ Charité - Universitätsmedizin Berlin, corporate member of Freie Universität Berlin, Humboldt-Universität zu Berlin, and Berlin Institute of Health, Department of Periodontology, Oral Medicine and Oral Surgery, 14197 Berlin, Germany.

^2^Department of Periodontology, University of Washington, Seattle, WA, USA

^3^ Charité - Universitätsmedizin Berlin, corporate member of Freie Universität Berlin, Humboldt-Universität zu Berlin, and Berlin Institute of Health, Institute for Biochemistry, 10117 Berlin, Germany.

^4^ Freie Universität Berlin, Institute of Pharmacy, Pharmacology and Toxicology, 14195 Berlin, Germany.

^5^ University of British Columbia, Faculty of Pharmaceutical Sciences, 2405 Wesbrook Mall, Vancouver, BC, V6T1Z3, Canada

^6^ Freie Universität Berlin, Institute for Chemistry and Biochemistry, 14195 Berlin, Germany.

^#^contributed equally

**Running title:** nanocarrier at oral mucosal sites

**Key words:** core-multishell nanocarrier, transepithelial resistance, penetration, oral mucosal equivalents

***Corresponding Author**

Prof. Dr. Henrik Dommisch

Department of Periodontology and Synoptic Dentistry Charité – Universitätsmedizin Berlin

Assmannshauser Straße 4-6

14197 Berlin, Germany

Phone: +49-(0)-30-450-562-322

Email: [henrik.dommisch@charite.de](mailto:henrik.dommisch@charite.de)

**Material and Methods**

**Franz cell experiments**

Penetration studies were performed as recently described [1]. Briefly, punched disks of porcine oral mucosal tissues (2 cm diameter) were mounted onto static-type Franz cells (diameter 7 mm, volume 5 ml, PermeGear, Bethlehem, PA, USA) with the epidermal part facing the air and the *lamina propria* having contact with the receptor fluid (phosphate buffered saline (PBS, pH 7.4). After 30 min, 91 μl/cm^2^ of the ICC-coupled CMS 10-E-15-350 were applied to the surface of the masticatory as well as the buccal mucosa for the time points indicated. Subsequently, the treated mucosal tissues were rinsed in PBS and subjected to immunohistochemical analyses.

To determine the attachment to the mucosal surfaces and to evaluate a potential penetration of the nanocarrier, tissues were embedded in tissue freezing medium (Jung, Nussloch, Germany) and stored at -80^o^C. Subsequently, microsections of 5 µm thickness were prepared using a freeze microtome (Frigocut 2800N, Leica, Bensheim, Germany). Attachment of the ICC-coupled CMS 10-E-15-350 and penetration into the tissue were analyzed by the use of the confocal laser scanning microscope LSM700MAT (CLSM, Zeiss, Jena Germany). For precise localization of the nanocarrier, the epidermis of the mucosal tissues was counterstained with a pan-cytokeratin antibody and the appropriate secondary antibody (both Invitrogen, Carlsbad, USA). Nuclei were visualized using Hoechst 33342 dye (Sigma-Aldrich, Steinheim, Germany). Images taken with the confocal laser scanning microscope were processed with the ZEN software (Zeiss, Jena, Germany). Three independent experiments were performed using three technical replicates for both masticatory and buccal mucosa at each time point.

**3D culture of *in vitro* organotypic mucosal equivalents**

To establish a model for tracking nanocarrier penetration within the oral mucosal tissue, a 3D organotypic cell culture was established [2]. Here, OKG4 cells and the fibroblast cell line (T0026) were co-cultured.

Briefly, 1 ml of a collagen I solution mixed with 4 × 10^5^ cells/ml gingival fibroblasts was transferred on an acellular collagen sheet from bovine type I collagen (0.77 mg/ml; Nutragen®, Advanced BioMatrix) in a Millicell® culture plate insert (30 mm diameter, pore size 0.4 µm; Merck). Inserts were placed in 6-well plates and incubated at 37°C and 5% CO_2_ for 1 h. The fibroblast-populated collagen-gels were cultured for 5 days in DMEM (Corning) containing 10% FCS (PAN-Biotech). The medium was then removed and 1 × 10^6^ OKG4 cells were added on top, and this co-culture was further incubated for 2 hours. Keratinocytes received DermaLife K medium containing 60 µM Ca^2+^ (Lifeline Cell Technology) via the inserts, while DMEM for fibroblasts was added to the well.

When the OKG4 had reached confluence, the mucosal equivalents were lifted to the air-liquid interface and cultured for another 8 days using DermaLife K medium with 1.4 mM Ca2^+^. During this period of time, half of the medium was changed every other day. To mimic the humidity and saliva, 100 µl of medium were added to the surface of the mucosal equivalent until the cell differentiation process was complete. Subsequently, the CMS 10-E-15-350 nanocarrier (50 µg/ml) was added to the mucosal equivalents to introduce a novel experimental design for penetration tracking. Co-cultures and penetration experiments (5 min penetration time) were repeated three times.

Following penetration and removal of excessive dyes through PBS washing, the mucosal equivalents were fixed in 4 % PFA(w/v)/0.025% saponin(w/v) (Carl Roth) in PBS for 4 h and subsequently subjected to embedding in paraffin or in tissue freezing medium (Leica Biosystems). 5 - 7 µm sections were produced and stained as described in the supplementary electronic file. Nanocarrier penetration was visualized using CLSM.

**Cell viability assays**

To determine a potential influence of the CMS 10-E-15-350 nanocarrier on gingival epithelial cells, MTT and sulphorhodamine B assays were performed using OKG4 cells (kindly provided by Susan Gibbs). For the MTT assay, 1 x 10^4^ cells were seeded into 96-well plates and were grown to 60-70% confluence. Subsequently, the culture medium was removed and replaced with 100 μl/well of fresh medium containing concentrations of the CMS nanocarrier as indicated for 24, 48 and 72 h. Then, 10µl MTT solution/well were added and the cells were incubated for 4 h at 37^o^C. After incubation, the supernatant was removed, the formazan crystals were dissolved by adding 100 µl acidic isopropanol to each well, and the colorimetric reaction was measured photometrically at 490 nm and quantified. Untreated cells served as a positive control and were set at 100% viable cells. Cells lysed with 4 % Triton X-100 before performing the MTT assay were taken as 0% viable cells. A total of three experiments were performed with each consisted of six technical replications. For further analysis, mean values of untreated controls cells were set at 100% in each of the three experiments. Test groups were normalized to untreated control cells. Graphs represent the three experimental sets, and error bars indicate standard deviations in the test groups when normalized to controls.

The sulphorhodamine B (SRB) assay is used for cell density determination, based on the measurement of cellular protein content. For this assay, OKG4 cells were grown to a confluence of 60-70% and were then exposed the concentrations of the CMS 10-E-15-350 nanocarrier as indicated for 24, 48 and 72 h. Subsequently, cell monolayers were treated with 10% trichloroacetic acid (w/v) and stained with 0.06% SRB (w/v) in 1% acetic acid for 30 min. Cells were then repeatedly washed using 1% acetic acid (v/v) followed by dissolution in 10 mM Tris (pH 10.5). Cell density was monitored using a microplate reader (Tecan, Gröding, Austria) at 492 nm. A total of three experiments were performed with each consisted of six technical replications. For further analysis, mean values of untreated controls cells were set at 100% in each of the three experiments. Test groups were normalized to untreated control cells. Graphs represent the three experimental sets, and error bars indicate standard deviations in the test groups when normalized to controls. Values >80% predicted no cytotoxic effects.

**Polymerase chain reaction (qPCR)**

OKG4 cells were cultured in 6-well tissue culture plates (2.5 x 10^5^ per well) 46 hours prior to stimulation experiments. After 30 hours, the medium was changed to medium containing high Ca^2+^-concentration (1.4 mM). 16 hours later, the cells were then stimulated for 3h, 6h or 12h using 5 µg/ml, 50 µg/ml and 500 µg/ml of the CMS nanocarrier.

Upon stimulation, cells were washed twice with PBS, and subsequently, cells were harvested using the lysis buffer (Qiagen, Hilden, Germany), and total RNA extraction was performed using the RNeasy Mini Kit (Qiagen) according to the manufacturer’s instructions [3-5]. An additional DNAse I treatment step was performed using the RNase free DNase I recombinant enzyme (Roche, Mannheim, Germany) followed by isopropanol precipitation of the RNA. Total RNA concentration was determined using the nanodrop technology (multi-plate reader, Thermo Fisher Scientific, Waltham, USA). Subsequently, the reverse transcription reaction (RT-PCR) was performed with an amount of 500 ng of total RNA using the High-Capacity cDNA Reverse Transcription Kit and oligo-(dT)-primers (Thermo Fisher Scientific) in full accordance with the manufacturer’s guidelines (using oligo-(dT)- and dNTP-primers instead of Random Primers).

Real-time PCR experiments were performed using the CFX Connect System (Bio-Rad, Hercules, USA) in combination with SYBR Select Master Mix (Thermo Fisher Scientific) according to the manufacturer’s instructions. The gene expression of TNF alpha, IL-1, IL-6 and IL-8 was normalized to the mRNA expression of β-Actin, and relative expression was calculated using the delta-delta ct method (Microsoft Excel, Redmond, WA, USA; GraphPad Prism Software, La Jolla, CA, USA). Primer Sequences were the following:

- Beta-actin (forward: CACGATGGAGGGGAAGACG; reverse: CACAGAGCCTCGCCTTTG);
- TNF alpha (forward: CCTGCTGCACTTTGGAGTGA; reverse: GAGGGTTTGCTACAACATGGG);
- IL-1 beta (forward: GAGCAACAAGTGGTGTTCTCC; reverse: AACACGCAGGACAGGTACAG);
- IL-6 (forward: ACAACCTGAACCTTCCAAAGA; reverse: GTTGGGTCAGGGGTGGTTAT);
- IL-8 (forward: AACTTCTCCACAACCCTCTG; reverse: TTGGCAGCCTTCCTGATT)

(metabion GmbH, Planegg/Steinkirchen, Germany). Control PCR reactions contained water instead of cDNA. Each experiment was performed with 6 technical replicates.

**ELISA experiments detecting IL-6 and IL-8**

Sandwich ELISAs (ImmunoTools) were performed to quantify cytokine concentrations. The medium from 6-well plates (Corning) was removed from the culture after 6, 24, or 72 h and stored at -20 °C until use. Samples were subjected to ELISA. The assay was performed in accordance with the manufacturer’s instructions using 100 µl of culture medium per well.

**Figure legends**

**Appendix figure 1**

Determination of cell growth of untreated control cells over time using (A) SRB and (B) MTT assays.

**Appendix figure 2**

Measurement of the metabolic activity (MTT) of primary gingival keratinocytes. Cells were exposed to nanocarrier concentrations of 50 and 500 µg/ml for 24 and 72 hours. Data were mean values of two different donors, each collected in technical quadruplicates. Two independent experiment were performed.

**Appendix figure 3**

(A) OKG4 cells in the presence of 60 µM and (B) 1.4 mM Ca^2+^ stained with Alexa Fluor 488-coupled wheat germ agglutinin (WGA; green).

**Appendix figure 4**

Z-Stack projection for visualization of the intracellular localization of the CMS 10-E-15-350 nanocarrier after 5 min, 30 min, and 6 hours. Intracellular perinuclear, but not intranuclear, localization was demonstrated following penetration.

**References**

1. Jager J, Obst K, Lohan SB, Viktorov J, Staufenbiel S, Renz H, Unbehauen M, Haag R, Hedtrich S, Teutloff C, Meinke MC, Danker K, Dommisch H (2018) Characterization of hyperbranched core-multishell nanocarriers as an innovative drug delivery system for the application at the oral mucosa. J Periodontal Res 53 (1):57-65. doi:10.1111/jre.12487

2. Dongari-Bagtzoglou A, Kashleva H (2006) Development of a highly reproducible three-dimensional organotypic model of the oral mucosa. Nat Protoc 1 (4):2012-2018. doi:10.1038/nprot.2006.323

3. Dommisch H, Chung WO, Jepsen S, Hacker BM, Dale BA (2010) Phospholipase C, p38/MAPK, and NF-kappaB-mediated induction of MIP-3alpha/CCL20 by Porphyromonas gingivalis. Innate Immun 16 (4):226-234. doi:1753425909339237 [pii]

10.1177/1753425909339237

4. Dommisch H, Chung WO, Rohani MG, Williams D, Rangarajan M, Curtis MA, Dale BA (2007) Protease-activated receptor 2 mediates human beta-defensin 2 and CC chemokine ligand 20 mRNA expression in response to proteases secreted by Porphyromonas gingivalis. Infect Immun 75 (9):4326-4333

5. Dommisch H, Reinartz M, Backhaus T, Deschner J, Chung W, Jepsen S (2012) Antimicrobial responses of primary gingival cells to Porphyromonas gingivalis. J Clin Periodontol 39 (10):913-922. doi:10.1111/j.1600-051X.2012.01933.x
