# Supplementary figures and images for: Characterization of an ester-based core-multishell (CMS) nanocarrier for the topical application at the oral mucosa
Source: Clin Oral Investig. 2021 Apr 5;25(10):5795–805. doi: 10.1007/s00784-021-03884-x (PMC8443517; doi:10.1007/s00784-021-03884-x)

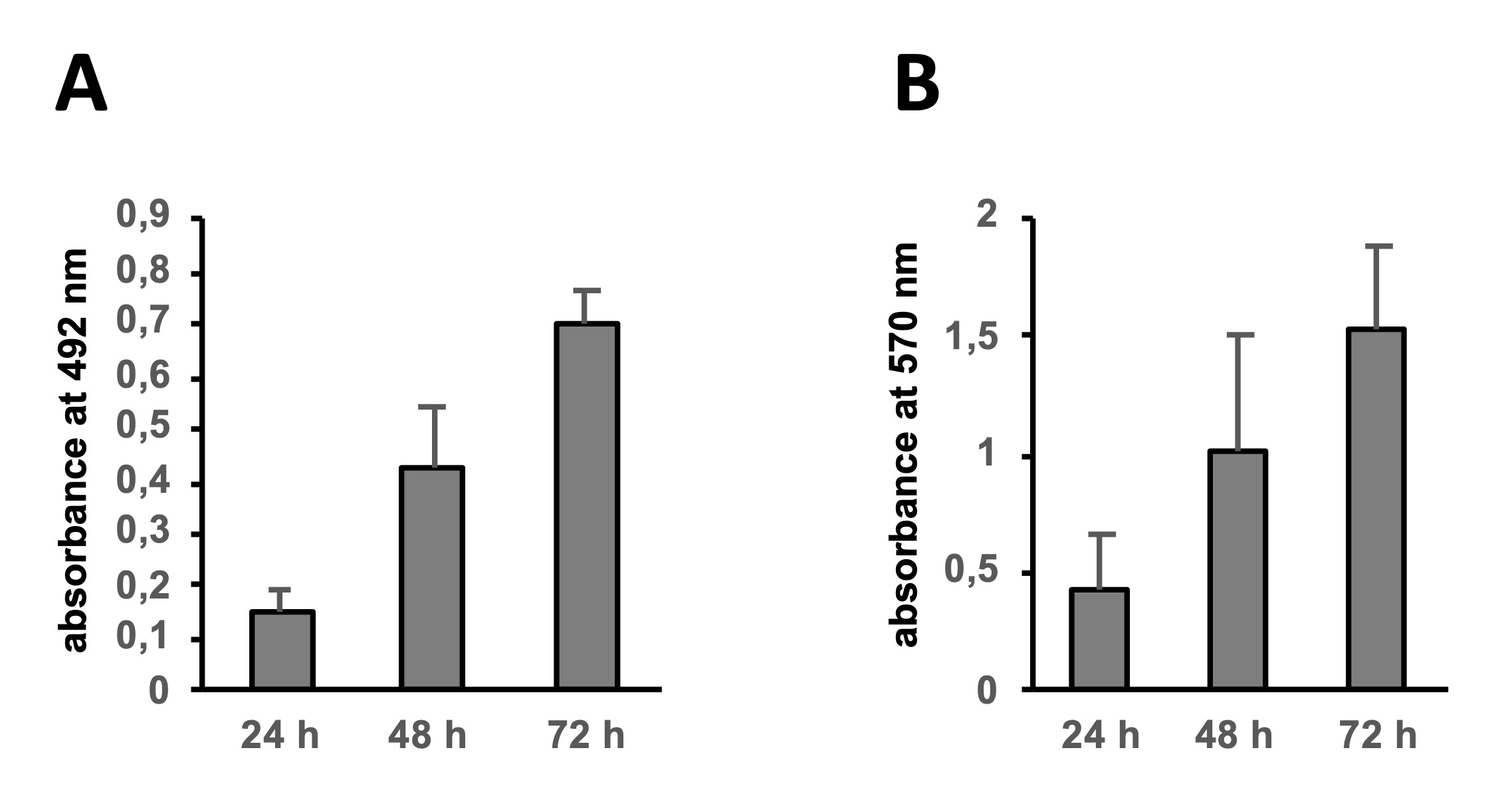

Supplement: Supplementary file 2 — (JPG 149 kb) [file 784_2021_3884_MOESM2_ESM.jpg]

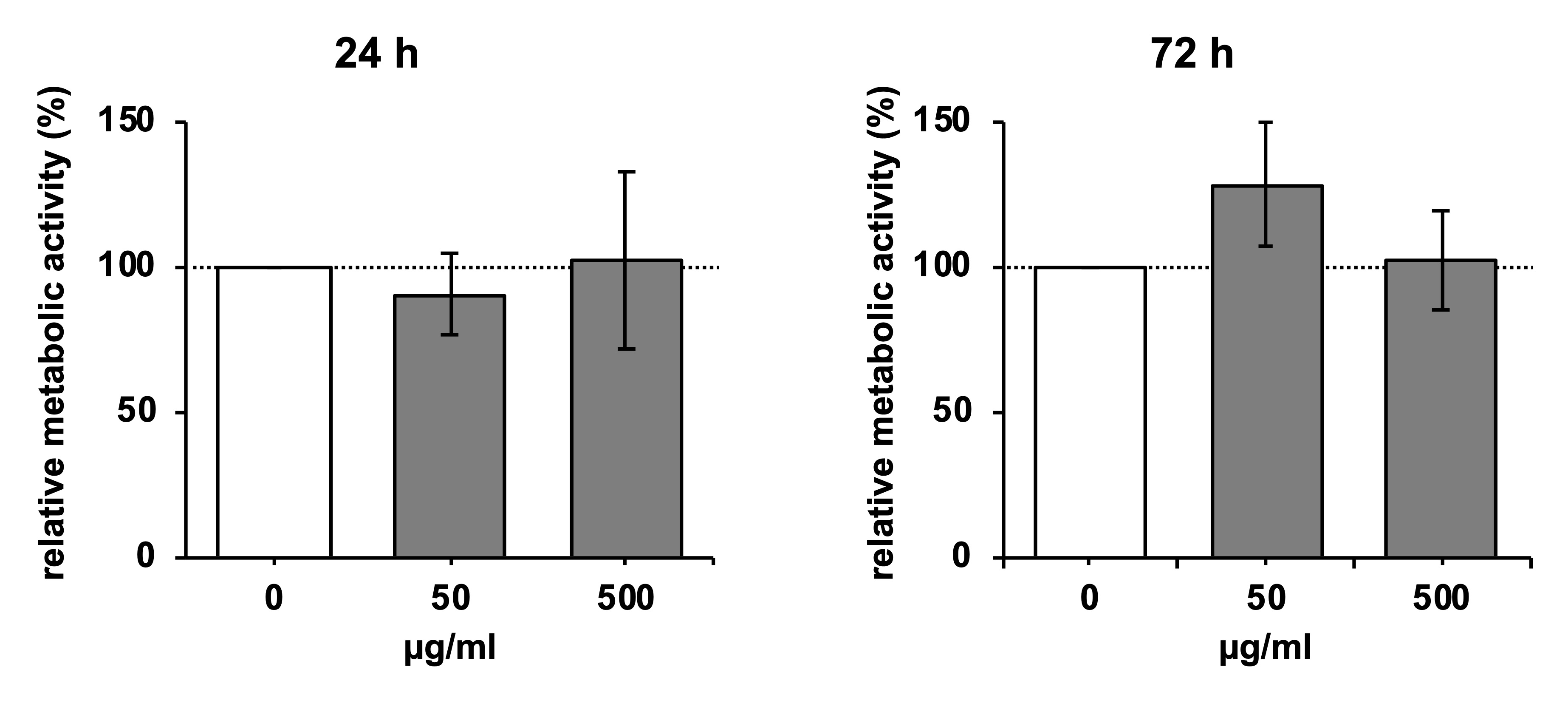

Supplement: Supplementary file 3 — (JPG 294 kb) [file 784_2021_3884_MOESM3_ESM.jpg]

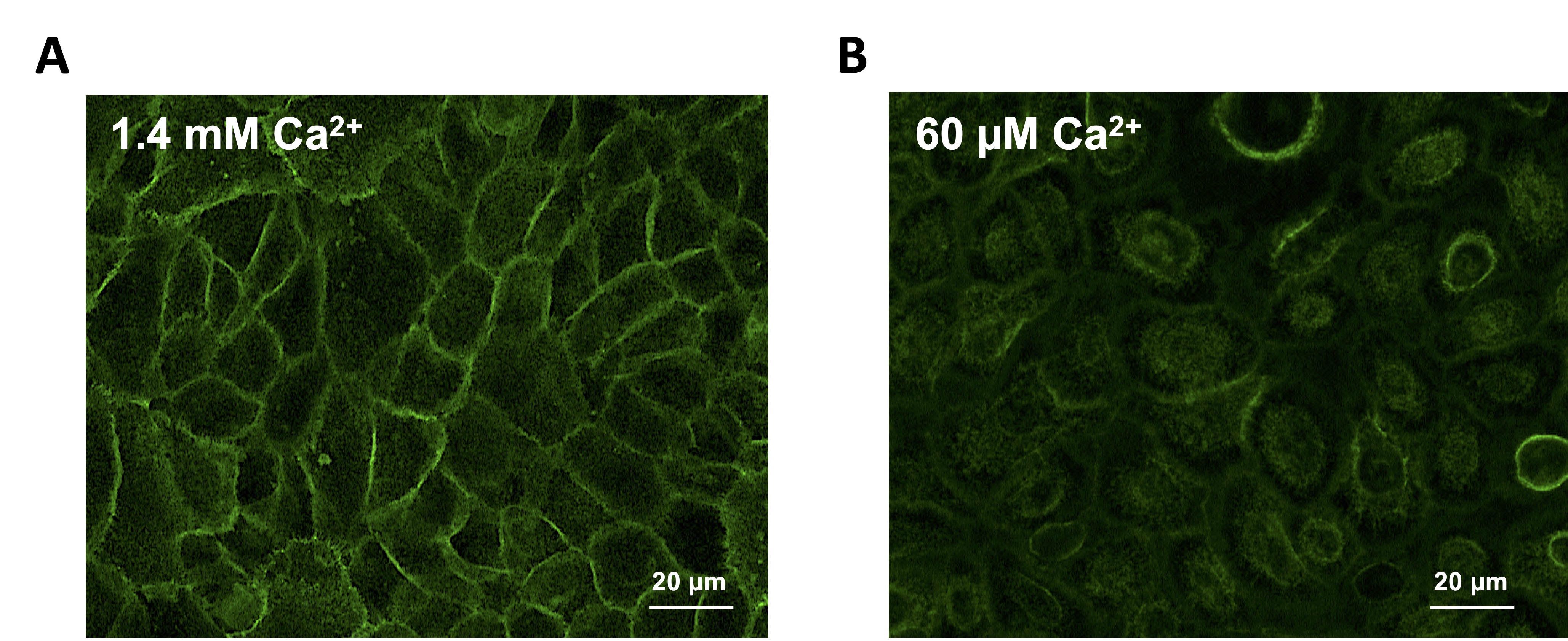

Supplement: Supplementary file 4 — (JPG 1317 kb) [file 784_2021_3884_MOESM4_ESM.jpg]

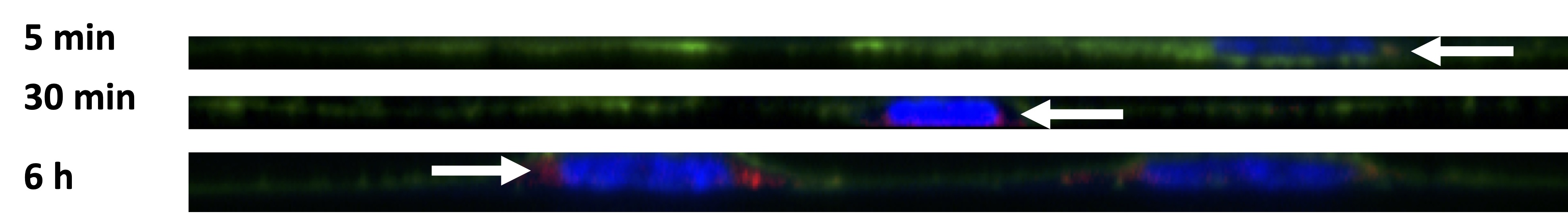

Supplement: Supplementary file 5 — (JPG 171 kb) [file 784_2021_3884_MOESM5_ESM.jpg]
